# The DLBCL90 gene‐expression assay identifies double‐hit lymphomas with high sensitivity in patients from two phase II clinical trials with high‐risk diffuse large B‐cell lymphoma

**DOI:** 10.1002/jha2.109

**Published:** 2020-12-05

**Authors:** Kathrine T. Isaksen, Klaus Beiske, Erlend B. Smeland, Judit Jørgensen, Marianne Brodtkorb, June Helen Myklebust, Mats Jerkeman, Leo Meriranta, Marja‐Liisa Karjalainen‐Lindsberg, Sirpa Leppä, David W. Scott, Harald Holte, Yngvild Nuvin Blaker

**Affiliations:** ^1^ Department of Cancer Immunology Institute for Cancer Research Oslo University Hospital Oslo Norway; ^2^ KG Jebsen Centre for B cell malignancies University of Oslo Oslo Norway; ^3^ Institute of Clinical Medicine University of Oslo Oslo Norway; ^4^ Department of Pathology Oslo University Hospital Oslo Norway; ^5^ Department of Hematology Aarhus University Hospital Aarhus Denmark; ^6^ Department of Oncology Oslo University Hospital Oslo Norway; ^7^ Department of Oncology Lund University and Skåne University Hospital Lund Sweden; ^8^ Helsinki University Hospital Comprehensive Cancer Centre and University of Helsinki Helsinki Finland; ^9^ iCAN Digital Precision Cancer Medicine Flagship Helsinki Finland; ^10^ Department of Pathology Helsinki University Hospital Helsinki Finland; ^11^ Centre for Lymphoid Cancer British Columbia Cancer Vancouver Canada

High‐grade B‐cell lymphoma with *MYC* and *BCL2* and/or *BCL6* rearrangements (HGBL‐DH/TH) comprise ∼8% of tumors with diffuse large B‐cell lymphoma (DLBCL) morphology [1] and is associated with poor outcome after standard R‐CHOP (rituximab, cyclophosphamide, doxorubicin, vincristine, and prednisone) therapy [2]. Accurate diagnosis is resource intensive as it requires fluorescence in situ hybridization (FISH) in all DLBCL tumors. Improved and facilitated diagnostics of the double‐hit population is needed to select these patients for new treatment approaches.

Recently, Ennishi and colleagues [3] developed a double‐hit gene‐expression signature (DHITsig) that identifies most double‐hit tumors defined by FISH in addition to high‐risk tumors with a similar gene‐expression profile. To train the DHITsig, they used HGBL‐DH/TH with *BCL2* rearrangement (HGBL‐DH/TH‐*BCL2*), which comprises the majority of HGBL‐DH/TH and displays a germinal center B‐cell‐like (GCB) phenotype [1]. The DHITsig was translated into an assay (DLBCL90) applicable to formalin‐fixed paraffin‐embedded (FFPE) biopsies, including the validated Lymph2Cx cell‐of‐origin‐ [4, 5] and Lymph3Cx primary mediastinal large B‐cell lymphoma signature assays [6]. In a population‐based cohort of 184 GCB‐DLBCL tumors, the DLBCL90 assay classified 23% of the tumors as DHITsig positive (DHITsig‐pos), 10% as DHITsig indeterminate (DHITsig‐ind), and 66% as DHITsig negative (DHITsig‐neg). The DHITsig‐pos and DHITsig‐ind groups were associated with inferior outcome after R‐CHOP therapy, and the combined DHITsig‐pos/ind group detected HGBL‐DH/TH‐*BCL2* with a sensitivity of 88% and specificity of 86% [3]. However, the assay has not yet been validated in a prospective clinical trial, and the prognostic value has not been investigated in patients treated with more intensive regimens than R‐CHOP.

The first objective of our study was to validate the sensitivity of the DLBCL90 assay, by detection of HGBL‐DH/TH‐*BCL2* in tumors with DLBCL morphology. Although not a perfect measurement of sensitivity, we expect that the broader defined DHITsig‐population comprises the majority of HGBL‐DH/TH‐*BCL2*. The second objective was to evaluate the prognostic significance of the DHITsig in patients treated with intensified therapy in a clinical trial.

We included patients with high‐risk de novo DLBCL from two Nordic phase II clinical trials [7, 8] with confirmed DLBCL NOS (WHO classification, 2008) and available FFPE tissue (n = 90). Patients were below 65 years of age and had age‐adjusted International Prognostic Index score 2‐3 and/or increased risk of CNS recurrence. In both trials, the patients received biweekly R‐CHOP with etoposide (R‐CHOEP‐14) and systemic CNS prophylaxis with HD‐Mtx and HD‐Ara‐C.

RNA was extracted from representative pre‐treatment FFPE tissue and digital gene expression was performed, applying the DLBCL90 assay on the NanoString platform (NanoString Technologies, Seattle, WA). The cutoff for DHITsig positivity was set between the DHITsig‐ind and DHITsig‐neg group as defined by Ennishi et al [3]. This was based on the optimal cutoff for the DHITsig, defined by the Youden Index, in a receiver operating curve of DHITsig versus HGBL‐DH/TH‐*BCL2* in a large independent cohort of DLBCL [9]. FISH break‐apart probes for *MYC*, *BCL2*, and *BCL6* were used to identify HGBL‐DH/TH. Translocation partner for *MYC* was investigated using FISH fusion probes for *MYC* and immunoglobulin heavy chain and FISH break‐apart probes for kappa and lambda light chains. Double protein expression by immunohistochemistry was scored by expert hematopathologists, and cell‐of‐origin assignment by immunohistochemistry was determined by Hans algorithm. Details on the patient cohort and experimental procedures are provided in Supporting Information. The data that support the findings in this study are available from the corresponding author upon request.

Successful molecular classification by the DLBCL90 assay was obtained for 89 of 90 cases. Three cases classified as primary mediastinal large B‐cell lymphoma were excluded from further analyses. For the remaining 86 cases, the DLBCL90 assay assigned 47 (55%) samples to the GCB‐subtype, 26 (30%) to the activated B‐cell like subtype, and 13 (15%) to the unclassified group. Patient characteristics were largely representative for the two study cohorts combined (Table S1).

The DHITsig was only detected in the GCB subtype, where 16 (34%) samples were assigned to the DHITsig‐pos group (including 11 DHITsig‐ind samples) (Figure 1). The DHITsig‐pos group had a higher median age than DHITsig‐neg GCBs (61 years vs 54 years, *P *= .005), otherwise no clinical characteristics differed between the two groups (Table S2).

**FIGURE 1:**
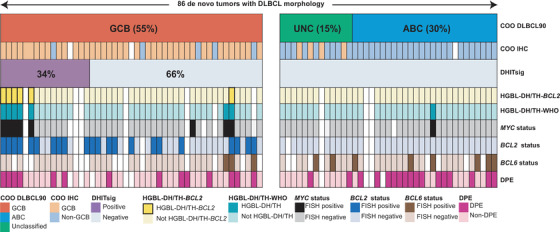
Performance of the DLBCL90 assay. The assay identified 86 DLBCL cases among 90 included lymphoma samples, and each column represents a patient specimen. The DLBCL90 assay assignment to cell‐of‐origin subtypes is shown in the first row and the DHITsig status in the third row. The samples are sorted according to the cell‐of‐origin subtype, as well as the percentage probability of belonging to the DHITsig‐pos group. The cell‐of‐origin subtype classification by immunohistochemistry and Hans algorithm is shown in the second row. Information regarding HGBL‐DH/TH defined by FISH, status for *MYC*, *BCL2, BCL6* rearrangements, and DPE‐status are shown below. Abbreviations: COO, cell‐of‐origin; UNC, unclassified group; ABC, activated B‐cell like subtype; IHC, immunohistochemistry; HGBL‐DH/TH‐*BCL2*, high‐grade B‐cell lymphoma with *MYC* and *BCL2* rearrangements with or without a *BCL6* rearrangement; HGBL‐DH/TH‐WHO, high‐grade B‐cell lymphoma with *MYC* and *BCL2* and/or *BCL6* rearrangements as defined by the WHO classification of 2016; *MYC* status, *MYC* rearrangement detected by FISH break‐apart probes; *BCL2* status, *BCL2* rearrangement detected by FISH break‐apart probes; *BCL6* status, *BCL6* rearrangement detected by FISH break‐apart probes; DPE, double protein expression of MYC and BCL2

FISH results were available from 77 samples, and six (8%) were identified as HGBL‐DH/TH‐*BCL2*, all in the GCB subtype. The DHITsig captured five of the six HGBL‐DH/TH‐*BCL2* (Figure 1). A sensitivity of 83% and a specificity of 89% for the DHITsig in detecting HGBL‐DH/TH‐*BCL2* are in line with previous findings [3] (Figure S1). Among the five HGBL‐DH/TH‐*BCL2* that were assigned to the DHITsig‐pos group, three had an immunoglobulin‐ and two had a non‐immunoglobulin translocation partner for *MYC*. The last HGBL‐DH/TH‐*BCL2* was a triple‐hit tumor assigned by the assay to the DHITsig‐neg group. This case had a non‐immunoglobulin translocation partner for *MYC*, and the patient had a favorable outcome with complete remission after first‐line therapy and was still in remission at the last follow‐up 45 months after diagnosis. Of note, none of the three HGBL‐DH/TH‐*BCL2* samples that were DHITsig‐neg in the study by Ennishi et al had any of the recurrent immunoglobulin translocation partners for *MYC*, and all patients had favorable outcomes [3]. These findings correspond with recent results described by the Lunenburg Lymphoma Biomarker Consortium [10]. Among the remaining 11 DHITsig‐pos cases, eight had available FISH results. Five of these displayed rearrangement of *BCL2*. Additionally, two of seven tested were double protein expressers (Figure 1).

The median follow‐up of living patients was 65 months. Within the GCB subgroup, there were no significant differences in outcome (OS, PFS) between the DHITsig‐pos and DHITsig‐neg group. In contrast to previous findings [3], the DHITsig‐pos group had excellent outcome, with a 5‐year PFS of 81% and 5‐year OS of 93% (Figure 2). Importantly, HGBL‐DH/TH determined by FISH also showed similar good outcome (Table S3). This was also observed in the combined total cohort from the two original trials [8].

**FIGURE 2:**
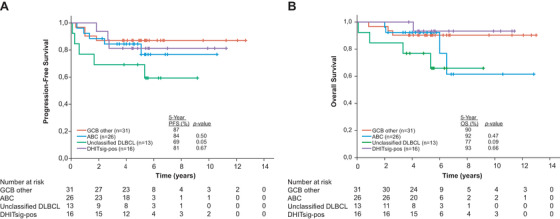
Prognostic significance of the DHITsig in young, high‐risk DLBCL patients treated with dose‐dense immunochemotherapy. A, Progression‐free survival for the DHITsig‐pos group versus the remaining DHITsig‐neg subgroups determined by the DLBCL90 assay. B, Overall survival for the DHITsig‐pos group versus the remaining DHITsig‐neg subgroups determined by the DLBCL90 assay. *P*‐values are derived from log‐rank tests comparing each group with the GCB other‐group (DHITsig‐neg GCBs)

A possible reason why we did not find a significantly inferior survival for DHITsig‐pos and HGBL‐DH/TH patients could be the limited sample size. Other reasons may be that the patients were relatively young (median age 55 years) and a selection bias for a more fit double‐hit patient population than those presented in population‐based cohorts. Additionally, our cohort included only HGBL‐DH/TH with DLBCL morphology, for which current evidence suggests better outcome than HGBL‐DH/TH with high‐grade morphology [10]. Furthermore, recent studies have shown that the adverse outcome of HGBL‐DH/TH is likely to be restricted to cases with an immunoglobulin translocation partner for *MYC* [10]. In our cohort, only three of the six HGBL‐DH/TH‐*BCL2* had an identified immunoglobulin translocation partner for *MYC*. Although the numbers are too small to conclude, all three cases achieved complete remission after first‐line therapy, and were still in remission at the last follow‐up 45, 68, and 84 months after diagnosis. Of importance, the intensive regimen may have contributed to the good outcome. This hypothesis is supported by the favorable results from the total trial cohort [8]. It is also in line with the current understanding that double‐hit tumors may benefit from an intensified treatment approach, although not shown in randomized trials [11].

Strengths of our study include uniformly treated patients from prospective clinical trials with long follow‐up. Additionally, FISH was performed in the majority of cases (90%). We do however acknowledge a limited sample size and findings should thus be interpreted with caution.

The DLBCL90 assay is a diagnostic tool that has potential for broad clinical application as it can be performed on routinely available FFPE specimens, and requires minimal hands‐on time. In contrast to FISH, which detects a limited number of genomic aberrations, the DLBCL90 assay operates on the gene‐expression level and the DHITsig may also capture more complex aberrations associated with the double‐hit phenotype. Importantly, the DHITsig also identifies true HGBL‐DH/TH cases missed by FISH [12]. A limitation with the assay is that it is not trained to detect HGBL‐DH with *BCL6* rearrangements, which have variable biology. Additionally, the proposed cutoff for the DHITsig between the DHITsig‐ind and DHITsig‐neg group includes more cases where the need for an intensified treatment approach is less clear.

Sha and colleagues [13] defined a molecular high‐risk group of DLBCL patients based on a Burkitt lymphoma‐like gene‐expression signature that likely overlaps with the DHITsig‐pos group. Furthermore, new taxonomies of DLBCL based on genetic alterations have also been proposed [14, 15]. Although these studies have greatly refined our understanding of the genetic heterogeneity in DLBCL, subgrouping based on several layers of genetic information is still a step away from the clinic.

In summary, we show that the DLBCL90 assay is feasible to run on routinely available FFPE specimens, with successful molecular classification in 99% of the cases. As expected, the DHITsig was only detected in the GCB subtype, where 34% of the tumors were classified as DHITsig‐pos. The DHITsig showed a high sensitivity of 83% in detecting HGBL‐DH/TH‐*BCL2*. In our cohort of young high‐risk patients treated with dose‐dense immunochemotherapy, the DHITsig and HGBL‐DH/TH status were not associated with inferior survival. However, the intensive regimen may have contributed to the favorable outcome [8]. Although our findings should be interpreted with caution due to small sample size, they support the current practice of giving intensified treatment to HGBL‐DH/TH tumors, and indicate that intensified treatment may also be advantageous for the broader DHITsig‐pos group. We anticipate that the DLBCL90 assay can help to identify high‐risk DLBCL populations in need of alternative treatment approaches, and include these patients in clinical trials to find the optimal treatment.

## AUTHOR CONTRIBUTIONS

H.H., E.B.S., Y.N.B., and K.T.I. designed the research study. H.H., S.L., J.J., and M.J. recruited patients and collected clinical data. K.B., M.K., L.M., Y.N.B., and K.T.I. performed the immunohistochemical and/or fluorescence in situ hybridization scoring. K.T.I., Y.N.B., H.H., E.B.S., M.B., J.H.M., and D.W.S. analyzed and interpreted the data. K.T.I. and Y.N.B. wrote the manuscript and generated all tables and figures. All co‐authors edited and approved the final manuscript.

## CONFLICT OF INTEREST

E.B.S.: Patents: named inventor on patents, one of which is licensed to NanoString. J.J.: Gilead: Membership on an entity's Board of Directors or advisory committees; Roche: Membership on an entity's Board of Directors or advisory committees. M.J.: Roche: Honoraria, Research Funding; Janssen: Honoraria, Research Funding; Gilead: Honoraria, Research Funding; Acerta: Honoraria, Research Funding; Celgene: Honoraria, Research Funding. S.L.: Research Funding: Amgen and Mundipharma related to the NLG‐LBC‐05 trial, Research funding and honoraria: Roche, Takeda, Bayer, Celgene and Janssen‐Cilag (not related to this study). D.W.S.: Research funding: Janssen, NanoString; Consulting: Celgene, Janssen, Abbvie; Patents: named inventor on patents, one of which is licensed to NanoString. H.H.: Novartis: Honoraria, Other: Advisory board: Gilead, Novartis, Takeda, Roche. For all remaining authors, there are no conflict of interest to disclose.

## Supporting information

Supporting information

## References

[1] Chapuy B , Stewart C , Dunford AJ , Kim J , Kamburov A , Redd RA , et al. Molecular subtypes of diffuse large B cell lymphoma are associated with distinct pathogenic mechanisms and outcomes. Nat Med. 2018;24:679‐90. 29713087 10.1038/s41591-018-0016-8 PMC6613387

[2] Ennishi D , Jiang A , Boyle M , Collinge B , Grande BM , Ben‐Neriah S , et al. Double‐hit gene expression signature defines a distinct subgroup of germinal center B‐cell‐like diffuse large B‐cell lymphoma. J Clin Oncol. 2019;37:190‐201. 30523716 10.1200/JCO.18.01583 PMC6804880

[3] Friedberg JW How I treat double‐hit lymphoma. Blood. 2017;130:590‐6. 28600333 10.1182/blood-2017-04-737320

[4] Hilton LK , Tang J , Ben‐Neriah S , Alcaide M , Jiang A , Grande BM , et al. The double‐hit signature identifies double‐hit diffuse large B‐cell lymphoma with genetic events cryptic to FISH. Blood. 2019;134:1528‐32. 31527075 10.1182/blood.2019002600 PMC6839951

[5] Holte H , Leppa S , Bjorkholm M , Fluge Ø , Jyrkkio S , Delabie J , et al. Dose‐densified chemoimmunotherapy followed by systemic central nervous system prophylaxis for younger high‐risk diffuse large B‐cell/follicular grade 3 lymphoma patients: results of a phase II Nordic Lymphoma Group study. Ann Oncol. 2013;24:1385‐92. 23247661 10.1093/annonc/mds621

[6] Leppa S , Jorgensen J , Tierens A , Meriranta L , Ostlie I , Brown PD , et al. Patients with high‐risk DLBCL benefit from dose‐dense immunochemotherapy combined with early systemic CNS prophylaxis. Blood Adv. 2020;4:1906‐15. 32380536 10.1182/bloodadvances.2020001518 PMC7218416

[7] Mottok A , Wright G , Rosenwald A , Ott G , Ramsower C , Campo E , et al. Molecular classification of primary mediastinal large B‐cell lymphoma using routinely available tissue specimens. Blood. 2018;132:2401‐05. 30257882 10.1182/blood-2018-05-851154 PMC6265647

[8] Petrich AM , Gandhi M , Jovanovic B , Castillo JJ , Rajguru S , Yang DT , et al. Impact of induction regimen and stem cell transplantation on outcomes in double‐hit lymphoma: a multicenter retrospective analysis. Blood. 2014;124:2354‐61. 25161267 10.1182/blood-2014-05-578963

[9] Rimsza LM . Gene expression‐based classifications: have they finally arrived? In: *Presentation at the 61st Annual American Society of Hematology Meeting in a Special Scientific Symposia Session entitled Changing the Taxonomy of Aggressive Lymphoma: Ready to Impact Clinical Management*?, Orlando, FL, 2019.

[10] Rosenwald A , Bens S , Advani R , Barrans S , Copie‐Bergman C , Elsensohn MH , et al. Prognostic significance of MYC rearrangement and translocation partner in diffuse large B‐cell lymphoma: a study by the Lunenburg Lymphoma Biomarker Consortium. J Clin Oncol. 2019;37:3359‐68. 31498031 10.1200/JCO.19.00743

[11] Schmitz R , Wright GW , Huang DW , Johnson CA , Phelan JD , Wang JQ , et al. Genetics and pathogenesis of diffuse large B‐cell lymphoma. N Engl J Med. 2018;378:1396‐407. 29641966 10.1056/NEJMoa1801445 PMC6010183

[12] Scott DW , King RL , Staiger AM , Ben‐Neriah S , Jiang A , Horn H , et al. High‐grade B‐cell lymphoma with MYC and BCL2 and/or BCL6 rearrangements with diffuse large B‐cell lymphoma morphology. Blood. 2018;131:2060‐64. 29475959 10.1182/blood-2017-12-820605 PMC6158813

[13] Scott DW , Mottok A , Ennishi D , Wright GW , Farinha P , Ben‐Neriah S , et al. Prognostic significance of diffuse large B‐cell lymphoma cell of origin determined by digital gene expression in formalin‐fixed paraffin‐embedded tissue biopsies. J Clin Oncol. 2015;33:2848‐56. 26240231 10.1200/JCO.2014.60.2383 PMC4554747

[14] Scott DW , Wright GW , Williams PM , Lih CJ , Walsh W , Jaffe ES , et al. Determining cell‐of‐origin subtypes of diffuse large B‐cell lymphoma using gene expression in formalin‐fixed paraffin‐embedded tissue. Blood. 2014;123:1214‐17. 24398326 10.1182/blood-2013-11-536433 PMC3931191

[15] Sha C , Barrans S , Cucco F , Bentley MA , Care MA , Cummin T , et al. Molecular high‐grade B‐cell lymphoma: defining a poor‐risk group that requires different approaches to therapy. J Clin Oncol. 2019;37:202‐12. 30523719 10.1200/JCO.18.01314 PMC6338391

