# Supplementary material for: The DLBCL90 gene‐expression assay identifies double‐hit lymphomas with high sensitivity in patients from two phase II clinical trials with high‐risk diffuse large B‐cell lymphoma
Source: EJHaem. 2020 Dec 5;2(1):104–8. doi: 10.1002/jha2.109 (PMC9175840; doi:10.1002/jha2.109)
Supplement: Supplementary file 1 — Supporting information [file JHA2-2-104-s001.pdf]

Supplementary materials

The DLBCL90 gene-expression assay identifies double-hit lymphomas with high sensitivity in patients from two phase II clinical trials with high-risk diffuse large B-cell lymphoma

Kathrine T. Isaksen[1-3], Klaus Beiske[3,4], Erlend B. Smeland[1,2], Judit Jørgensen[5], Marianne Brodtkorb[1,2], June Helen Myklebust[1,2], Mats Jerkeman[7], Leo Meriranta[8,9], Marja-Liisa Karjalainen-Lindsberg[10], Sirpa Leppä[8,9], David W. Scott[11], Harald Holte[2,6] and Yngvild Nuvin Blaker[1-3]

[1]Department of Cancer Immunology, Institute for Cancer Research, Oslo University Hospital, Oslo, Norway
[2]KG Jebsen Centre for B cell malignancies, University of Oslo, Oslo, Norway
[3]Institute of Clinical Medicine, University of Oslo, Oslo, Norway
[4]Department of Pathology, Oslo University Hospital, Oslo, Norway
[5]Department of Hematology, Aarhus University Hospital, Aarhus, Denmark
[6]Department of Oncology, Oslo University Hospital, Oslo, Norway
[7]Department of Oncology, Lund University and Skåne University Hospital, Lund, Sweden
[8]Helsinki University Hospital Comprehensive Cancer Centre and University of Helsinki, Helsinki, Finland
[9]iCAN Digital Precision Cancer Medicine Flagship, Helsinki, Finland
[10] Helsinki University Hospital, Department of Pathology, Helsinki, Finland
[11]Centre for Lymphoid Cancer, British Columbia Cancer, Vancouver, Canada.

1) Study cohorts

All patients included in our study were treated in two prospective, Nordic phase II trials for young, clinically high-risk diffuse large B-cell lymphoma (DLBCL) patients (NLG-LBC-04 trial and NLG-LBC-05 trial) (Holte, *et al* 2013; Leppa, *et al* 2020). In both trials, inclusion criteria comprised age 18-64 years, previously untreated and histologically confirmed CD20-positive DLBCL or follicular lymphoma (FL) grade 3B, ECOG status ≤3 and age-adjusted IPI 2-3. In the NLG-LBC-05 trial, DLBCL patients with site-specific risk factors for CNS recurrence could also be included. Increased risk of CNS recurrence was defined as more than one extranodal site, testicular lymphoma stage IIE and higher, paranasal sinus and orbital lymphoma with destruction of bone, or large cell infiltration of the bone marrow. In both trials, patients received biweekly rituximab, cyclophosphamide, doxorubicin, vincristine, prednisone and etoposide (R-CHOEP-14), and systemic CNS prophylaxis with HD-Mtx and HD-Ara-C. CNS prophylaxis was given at the start of treatment in the NLG-LBC-05 trial, and

at the end of treatment in the NLG-LBC-04 trial. Patient characteristics were comparable between the two trials, with the exception of more patients with > 1 extranodal site in the NLG-LBC-05 trial (data not shown). Five-year progression-free survival was superior in the NLG-LBC-05 trial compared to the NLG-LBC-04 trial, while there was no difference in 5-year overall survival between the two trials (Leppa, *et al* 2020). The protocols were approved by the medical agencies and ethics committees in Norway, Finland, Denmark and Sweden. All patients with confirmed DLBCL NOS (WHO classification, 2008) and available formalin-fixed paraffin-embedded (FFPE) tissue that yielded sufficient RNA for the NanoString assay were selected for this study (n=90).

2) Digital gene expression

RNA was extracted from 90 formalin-fixed paraffin-embedded (FFPE) tissue biopsies using the Qiagen AllPrep FFPE kit (Qiagen, Hilden, Germany). Digital gene expression was performed on 200 ng RNA from the majority of cases (n= 77), applying the DLBCL90 assay on the NanoString platform (NanoString Technologies, Seattle, WA). In twelve cases the RNA input was 140 ng, and in one case 117 ng.

3) Fluorescence *in situ* hybridization (FISH) and immunohistochemical (IHC) analyses

FISH dual-color break-apart probes for *MYC* (1N63, Abbott Vysis), *BCL2* (5N51, Abbott, Vysis) and *BCL6* (1N23, Abbott Vysis) were used for identification of High-grade B-cell lymphoma with *MYC* and *BCL2* and/or *BCL6* rearrangements (HGBL-DH/TH). Hybridized paraffin sections were evaluated in a Zeiss ImagerZ2 fluorescence microscope applying Z-stack imaging and the ISIS software from MetaSystems (Altlussheim, Germany). In representative areas, the percentage distribution of single color and fused color fluorescence signals were calculated in 80-100 nuclei, excluding internuclear and non-specific signals. Samples were reported positive when the percentage of specific intranuclear single color signals exceeded 15%. For the 6 HGBL-DH/TH-*BCL2* tumors, translocation partner for *MYC* was investigated using FISH fusion probes for *MYC* and immunoglobulin heavy chain (IGH) (4N10, Abbott Vysis). In case of negative results, further testing was performed using FISH break-apart probes for kappa (XL 2p11 IGK BA Break Apart Probe, Order NumberD-5116-100-OG, Metasystems) and lambda light chains (XL 22q11 IGL BA Break Apart Probe, Order NumberD-5117-100-OG, Metasystems). Additionally, 2 of the cases were investigated using the XL MYC BA Triple-color Break Apart Probe (Order NumberD-6030-100-TC, Metasystems), which allows the identification of the breakpoint location proximal or distal of

C-MYC on chromosome 8. For the 5 HGBL-DH/TH-*BCL2* cases that were classified as DHITsig-pos by the DLBCL90 assay, 2 used IGH genes as translocation partner for *MYC* as demonstrated by the C-MYC-IGH fusion probes. Additionally, 1 case showed rearrangement of IGL genes. This suggests the presence of a t(8;22), which was further supported by the location of the breakpoint proximal of *C-MYC* on chromosome 8. For the 2 remaining DHITsig-pos cases, no immunoglobulin translocation partner for *MYC* was detected. The sixth HGBL-DH/TH-*BCL2* was a triple hit tumor that was classified as DHITsig-neg by the DLBCL90 assay. In this case no immunoglobulin translocation partner for *MYC* was detected.

Double protein expression (DPE) was assessed by immunohistochemical (IHC) staining of BCL2 (clone BCL-2/100/D5, Novocastra) and MYC (ab32072, clone Y69, Abcam) and scored by expert hematopathologists with a 50% cut-off for BCL2 and 40% cut-off for MYC. Cell-of-origin (COO) assignment by IHC was determined by Hans algorithm (Hans, *et al* 2004) using antibodies for CD10, BCL6 and MUM1.

4) Statistical analyses

The Kaplan Meier method was used to estimate progression-free survival (PFS) and overall survival (OS), and log-rank test was performed to compare groups. OS was defined as time from diagnosis to death or last follow-up. Patients alive at last follow-up were censored. PFS was defined as time from diagnosis to disease relapse, progression or death due to any cause. Fisher's exact test was used for comparison of categorical patient characteristics and Mann-Whitney U test was used for comparison of continuous variables. Univariable Cox proportional hazard regression models were used to analyze potential prognostic factors in the GCB subgroup. All tests were to-sided, and a threshold of 0.05 was used for significance. All analyses were performed using SPSS software version 25.0 (IBM, Armonk, NY, USA).

5) Performance of the DLBCL90 COO module (Lymph2Cx) and Hans algorithm

The DLCBL90 assay includes the Lymph2Cx assay, which is a recognized gene-expression profiling assay for COO. The maintained accuracy of the DLBCL90 COO assignment compared with the original Lymph2Cx assay is shown by Ennishi and colleagues (Ennishi, *et al* 2019). In our cohort, the DLBCL90 COO module assigned 47 (55%) samples to the GCB-subtype, 26 (30%) to the activated B-cell like (ABC) subtype and 13 (15%) to the unclassified group. Subtype annotation by IHC based on Hans algorithm was available for 83 samples, and the concordance with COO assignment by DLBCL90 was 94% (Figure 1). COO was not

associated with patient outcome, either using the Hans algorithm, or the DLBCL90 assay. However, the 13 patients assigned to the unclassified group by the DLBCL90 assay had inferior outcome compared to the patients assigned to the GCB subtype (5-year PFS: 69% vs 85%, $p = 0.04$; 5-year OS: 77% vs 91% $p = 0.03$) (Supplemental Figure 2 and 3).

6) Double protein expression and prognostic factors in the GCB subtype

MYC- and BCL2 protein expression status was available for 77 samples, and 31 (40%) were identified as DPE lymphomas. Within the GCBs, DPE was not associated with the DHITsig-pos group. This could be due to the limited sample size.

In the GCB subtype, ECOG performance status ≥2 was the only feature associated with inferior 5-year PFS (94% vs 62%; HR=7.03, 95% CI 1.36-36.3, $p=0.02$). Otherwise, no clinical or biological variables were associated with inferior 5-year PFS or OS in the GCB subtype (Supplemental Table 3).

7) Supplementary references

Ennishi, D., Jiang, A., Boyle, M., Collinge, B., Grande, B.M., Ben-Neriah, S., Rushton, C., Tang, J., Thomas, N., Slack, G.W., Farinha, P., Takata, K., Miyata-Takata, T., Craig, J., Mottok, A., Meissner, B., Saberi, S., Bashashati, A., Villa, D., Savage, K.J., Sehn, L.H., Kridel, R., Mungall, A.J., Marra, M.A., Shah, S.P., Steidl, C., Connors, J.M., Gascoyne, R.D., Morin, R.D. & Scott, D.W. (2019) Double-Hit Gene Expression Signature Defines a Distinct Subgroup of Germinal Center B-Cell-Like Diffuse Large B-Cell Lymphoma. *Journal of Clinical Oncology,* **37,** 190-201.

Hans, C.P., Weisenburger, D.D., Greiner, T.C., Gascoyne, R.D., Delabie, J., Ott, G., Muller-Hermelink, H.K., Campo, E., Braziel, R.M., Jaffe, E.S., Pan, Z., Farinha, P., Smith, L.M., Falini, B., Banham, A.H., Rosenwald, A., Staudt, L.M., Connors, J.M., Armitage, J.O. & Chan, W.C. (2004) Confirmation of the molecular classification of diffuse large B-cell lymphoma by immunohistochemistry using a tissue microarray. *Blood,* **103,** 275-282.

Holte, H., Leppa, S., Bjorkholm, M., Fluge, Ø., Jyrkkio, S., Delabie, J., Sundstrom, C., Karjalainen-Lindsberg, M.L., Erlanson, M., Kolstad, A., Fossa, A., Østenstad, B., Lofvenberg, E., Nordstrom, M., Janes, R., Pedersen, L.M., Anderson, H., Jerkeman, M. & Eriksson, M. (2013) Dose-densified chemoimmunotherapy followed by systemic central nervous system prophylaxis for younger high-risk diffuse large B-cell/follicular grade 3 lymphoma patients: results of a phase II Nordic Lymphoma Group study. *Annals of Oncology,* **24,** 1385-1392.

Leppa, S., Jorgensen, J., Tierens, A., Meriranta, L., Ostlie, I., Brown, P.D., Fagerli, U.M., Larsen, T.S., Mannisto, S., Munksgaard, L., Maisenholder, M., Vasala, K., Meyer, P., Jerkeman, M., Bjorkholm, M., Fluge, O., Jyrkklo, S., Liestol, K., Ralfkiaer, E., Spetalen, S., Beiske, K., Karjalainen-Lindsberg, M.L. & Holte, H. (2020) Patients

with high-risk DLBCL benefit from dose-dense immunochemotherapy combined with early systemic CNS prophylaxis. *Blood Advances*, **4,** 1906-1915.

8) Supplementary tables

Supplemental Table 1. Patient characteristics of the 86 patients in the present DLBCL90-study vs the total cohort in the two clinical trials

Characteristics	DLBCL90 cohort (n=86)	Total cohort (n= 295)	*p*
**Age, years, n (%)**			
Median (range)	55 (22-65)	54 (18-65)	0.335
< 60 years	58 (67)	207 (70)	
> 60 years	28 (33)	88 (30)	0.629
**Sex, n (%)**			
Male	57 (66)	185 (63)	
Female	29 (34)	110 (37)	0.545
**Stage, n (%)**			
I, II	8 (9)	17 (6)	
III, IV	78 (91)	278 (94)	0.243
**LDH, n (%)**			
Normal	8 (9)	17 (6)	
Elevated	78 (91)	278 (94)	0.243
**ECOG, n (%)**			
0-1	54 (63)	201 (68)	
≥ 2	32 (37)	94 (32)	0.354
**Extranodal sites, n (%)**			
0-1	32 (37)	155 (53)	
≥ 2	54 (63)	140 (48)	**0.012**
**B-symptoms, n (%)**			
No	35 (41)	113 (38)	
Yes	51 (59)	182 (62)	0.689
**aaIPI score, n (%)**			
0-2	55 (64)	210 (71)	
3	31 (36)	85 (29)	0.200

NOTE. Boldface indicates significance.
Abbreviations: LDH: lactate dehydrogenase;
ECOG: Eastern Cooperative Oncology Group performance status;
aaIPI: age-adjusted International Prognostic Index

Supplemental Table 2: Patient characteristics DHITsig-pos group vs. GCB other

Characteristics	DHITsig-pos (n=16)	GCB other (n = 31)	*p*
**Age, years, n (%)**			

Median (range)	61 (46-65)	54 (22-63)	**0.005**
< 60 years	7 (44)	25 (81)	
> 60 years	9 (56)	6 (19)	**0.019**
**Sex, n (%)**			
Male	7 (44)	23 (74)	
Female	9 (56)	8 (26)	0.057
**Stage, n (%)**			
I, II	1 (6)	1 (3)	
III, IV	15 (94)	30 (97)	1.000
**LDH, n (%)**			
Normal	1 (6)	2 (7)	
Elevated	15 (94)	29 (94)	1.000
**ECOG , n (%)**			
0-1	10 (63)	24 (77)	
≥ 2	6 (38)	7 (23)	0.318
**Extranodal sites, n (%)**			
0-1	5 (31)	10 (32)	
≥ 2	11 (11)	21 (68)	1.000
**B-symptoms, n (%)**			
No	8 (50)	14 (45)	
Yes	8 (50)	17 (55)	0.768
**aaIPI score, n (%)**			
0-2	9 (56)	25 (81)	
3	7 (44)	6 (19)	0.096
***MYC*-TR, n (%)**			
No	7 (58)	23 (89)	
Yes	5 (42)	3 (11)	0.081
NA	4	5	
***BCL2*-TR, n (%)**			
No	3 (23)	16 (57)	
Yes	10 (77)	12 (43)	0.052
NA	3	3	
**HGBL-DH/TH-BCL2, n (%)**			
No	8 (62)	27 (96)	
Yes	5 (38)	1 (4)	**0.008**
NA	3	3	
**DPE, n (%)**			
No	8 (57)	20 (71)	
Yes	6 (43)	8 (29)	0.49
NA	2	3	

NOTE. Boldface indicates significance.
Abbreviations: LDH: lactate dehydrogenase;
ECOG: Eastern Cooperative Oncology Group performance status;
aaIPI: age-adjusted International Prognostic Index;
NA: not available; TR: translocations

Supplemental Table 3: Univariate cox regression analyses in the GCB subtype

	Progression-free survival		Overall Survival	
**Variables**	**HR (95% CI)**	***p***	**HR (95% CI)**	***p***
DHIT signature (pos/ind vs neg)	1.38 (0.31-6.18)	0.67	0.60 (0.06-5.77)	0.66
HGBL-DH/TH-*BCL2* (pos vs neg)	1.39 (0.16-12.4)	0.77	0.04 (0.00->100)	0.64
DPE (pos. vs neg.)	0.77 (0.15-3.97)	0.76	0.68 (0.07-6.60)	0.74
Age (≥60 vs <60 years)	1.63 (0.36-7.27)	0.53	0.03 (0.00-155)	0.41
Stage (III/IV vs I/II)	21.5 (0.00->100)	0.70	21.5 (0.00->100)	0.77
LDH (elevated vs normal)	0.39 (0.05-3.24)	0.38	0.17 (0.02-1.70)	0.13
ECOG performance status (≥2 vs 0-1)	7.03 (1.36-36.3)	**0.02**	7.84 (0.82-75.3)	0.08
Extranodal sites (>1 vs 0-1)	0.30 (0.07-1.37)	0.12	0.44 (0.06-3.12)	0.41
age-adjusted IPI (3 vs 0-2)	1.90 (0.42-8.46)	0.40	0.80 (0.08-7.66)	0.84
B symptoms (yes vs no)	2.17 (0.42-11.2)	0.35	2.52 (0.26-24.3)	0.42
Ki67 (≥80 vs <80)	1.16 (0.23-5.73)	0.86	3.55 (0.37-34.1)	0.27
HGBL-DH/TH (pos vs neg)	1.12 (0.13-10.0)	0.92	0.04 (0.00->100)	0.61
Gender (male vs female)	3.76 (0.45-31.2)	0.22	1.74 (0.18-16.7)	0.63

9) Supplementary Figures

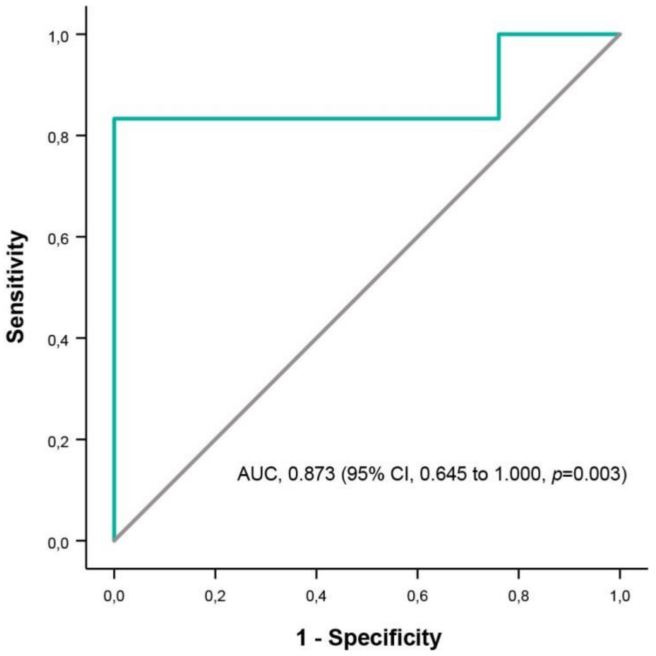

Supplemental Figure 1. Receiver operating characteristic curve (ROC) for the DHITsig score versus HGBL-DH/TH-*BCL2* as outcome of interest. Applying the threshold for

DHITsig-pos versus DHITsig-neg as defined in Ennishi et al(Ennishi, *et al* 2019) (DHITsig score -15,6), the sensitivity and specificity of the test is 83% and 89%, respectively. AUC, Area under the curve.

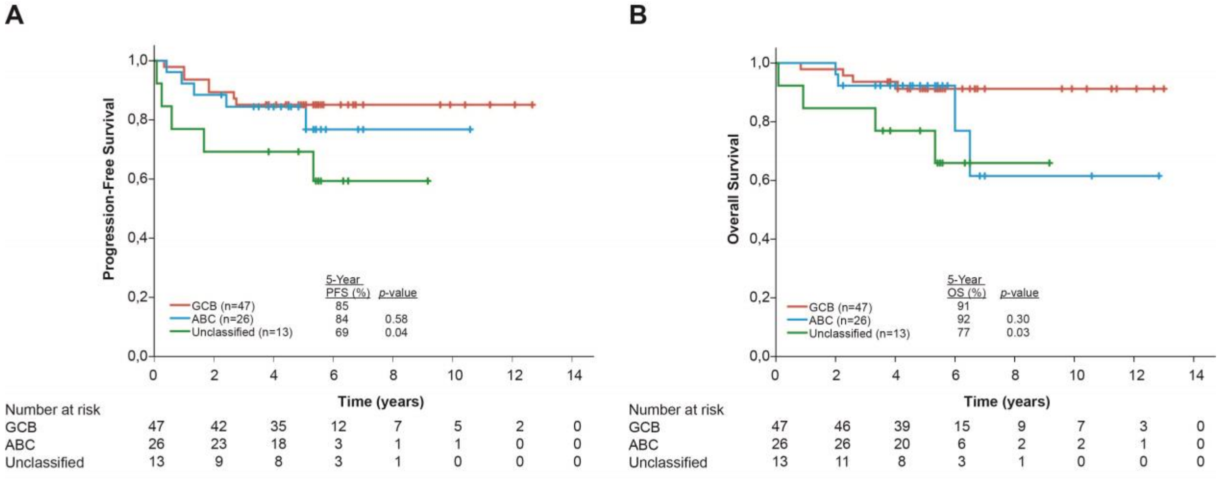

Supplemental Figure 2. Prognostic significance of COO determined by the DLBCL90 assay. (A) Progression-free survival for COO subtypes determined by the DLBCL90 assay. (B) Overall survival for COO subtypes determined by the DLBCL90 assay. P-values are derived from log-rank tests comparing each group with the GCB-group (including the DHITsig-pos group)

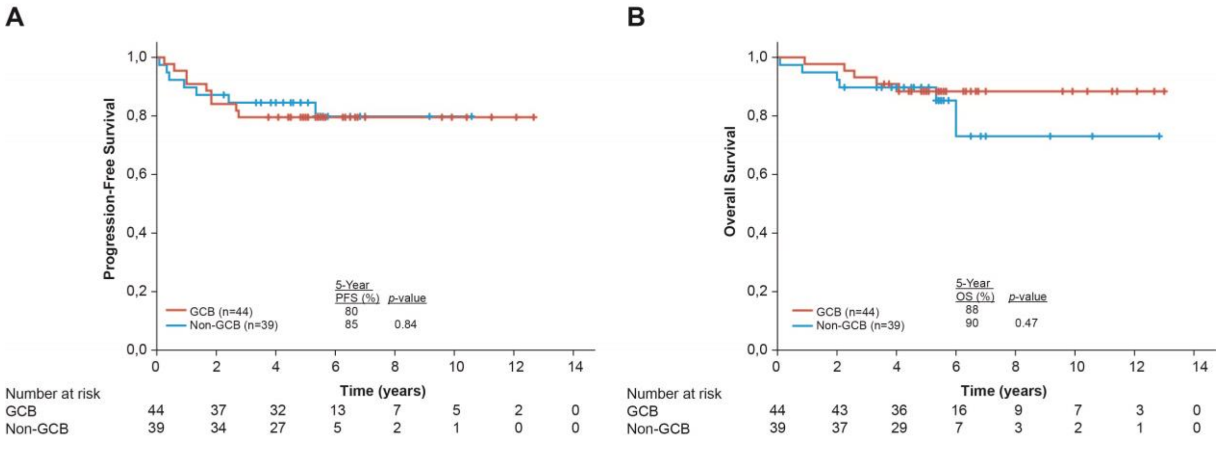

Supplemental Figure 3. Prognostic significance of COO determined by Hans algorithm.

(A) Progression-free survival for COO subtypes determined by Hans algorithm. (B) Overall survival for COO subtypes determined by Hans algorithm. P-values are derived from log-rank tests.
